# Chloroform in Surgery and Midwifery

**Published:** 1848-04

**Authors:** Charles H. Cragin

**Affiliations:** Georgetown, D. C.


*Chloroform in Surgery and Midwifery.* By CHARLES H. CRAGIN, A. M., M. D., Georgetown, D. C.

Having used this agent in several operations, and also a few times in medical cases, I offer the following as the result. My first case occurred in a boy ten years old, who had been attacked the previous winter with acute inflammation of the right fibula, which resulted in necrosis. The boy was in other respects in excellent health. I placed the patient under the influence of chloroform in about two minutes. At first he struggled and complained of being strangled, probably from its being used too rapidly and in too concentrated a state; but at last he lay quiet and snored through the operation, which lasted some half hour before the wound was dressed and he placed in bed. Not the slightest manifestation of pain or consciousness was shown throughout. I noticed considerable rumbling in his bowels, and after all was over, though he lost but little blood, his pulse fell and became alarmingly small and feeble. We opened the doors, exhibited spts. ammoniæ, applied cold water to his face with a dash, when he tried to vomit, and soon threw up his breakfast. After taking a teaspoonful or two of strong brandy he revived, but did not become fully awake before an hour or more. I used in this case doubtless too much chloroform, as the gentleman who had charge of the handkerchief threw on drachm after drachm, and kept it constantly applied to the face. In all, nearly an ounce was used. I remained until his pulse regained its force, and no accident subsequently occurred to the boy, who complained the next day of no headache, but of being starved. He is now running about and quite well. It is worthy of remark, that this boy has a younger sister who has caries of the right tibia very close to the knee-joint, and that he lost an older sister some two years since, before I knew the family, with some disease of the bones.

CASE II.—A married female, aged 24, two weeks after her confinement, sent to consult me about her arm. I found the left humerus much swelled a little below the middle of the bone, tender on pressure, paining her at all times, but at night greatly aggravated. She had suffered thus from childhood, and had exhausted all means that medicine could offer without avail. I proposed to her to take the chloroform, and then I would open the bone with a trephine, and perhaps I might find abscess there as the cause of her sufferings; and if not, the discharge from the vessels of the bone and the soft parts, aided by the internal administration of iodide of potassium, might effect a cure. To this she assented. It required nearly three drachms of chloroform to soporize her. At last, supposing her asleep, I commenced my incision through the soft parts, when I was interrupted by a scream from my patient. I stopped a few seconds till she breathed in a snoring manner, and then completed the operation. Pus was discharged and the cavity kept open with lint, and the wound dressed, when she awoke. She seemed confused for a few seconds, but complained of nothing; she recollected my first cut with the knife, but all the rest was unfelt. She, too, had not the slightest ill result, but is entirely relieved from pain, and the wound discharges less and less; she says she is better than she has been since she could recollect. The pulse in this case was, from mental anxiety, probably, about 100, but as soon as she became soporized it fell to 72, and soon to 45, where it remained full and of some force till the moment she awoke, when it rose again rapidly. She awoke without any stimulus.

Case III.—I removed a tumour from the shoulder (immediately over the joint) of a coloured woman in good health, aged 37. She tried hard to remove the handkerchief from her mouth, and rose several times from the chair, but finally fell asleep after inhaling three or four drachms of the chloroform. I soon dissected out the tumour, which was fatty, and enclosed in a cyst about three inches in diameter and one and a half thick. Her pulse soon fell to 35, (but full,) where it remained for some minutes, then very gradually rose to its natural standard. She was talking of a variety of things during the operation, without any signs of suffering; said she was "safe, as Christ was at the helm," &c. She gradually awoke while the wound was being washed and exposed to the air to arrest hemorrhage, and asked "why I didn't take out the tumour." After some time, without any further use of chloroform, I proceeded to unite the flaps in the integuments by several sutures, some of which she felt, but most of which she did not. I showed her the tumour, which she felt and looked at for some time, but asked again, in a few minutes, "what I was waiting for? she wanted that tumour out." I again showed it to her, and she was surprised that it was taken out without her knowledge, but soon forgot it again, and asked me to "make haste and take the tumour out of her shoulder." Does not this incident prove that as the eye saw distinctly, and it was forgotten a moment afterwards, so pain may be actually felt when sleep is produced but entirely forgotten when awake? She was a little sick at the stomach when she woke, but since has had no unpleasant feeling or effect whatever from the chloroform. The wound is healed well.

Case IV.—I removed a painful warty growth from the back of the neck of a lady, aged 35, and in good health. Instead of sleeping after inhaling the chloroform, the muscles of the neck became rigid and her tongue loose, so that it went as rapidly as it well could. After three or four strokes of the knife, I removed the wart without any signs of suffering on her part. She still talked incoherently for some minutes, but at last awoke. She said she had seen a great flame, and had passed by this to heaven, where she had been all the time. She was much surprised when I showed her what I had removed from her neck during her eloquence. She soon complained of nausea and vomited, but after lying down a little while she recovered entirely. Her pulse was natural throughout, and she has had no bad symptom since, except loss of appetite for a day or two. I learned upon inquiry that laudanum affected her in much the same manner as did the chloroform.

Case V.—I performed the operation for absorption on both eyes of a coloured man, aged 56, affected with soft cataract. I had great difficulty in getting him under the influence of chloroform, as he breathed the fifth drachm before he became quiet and insensible. It seemed to irritate his lungs, exciting a cough. At length he got up, refusing to take any more of the "bad stuff," but finally yielded and fell asleep. The pupils had been previously dilated by belladonna, but they seemed to contract slightly from the chloroform; the operation was soon completed without any sign of pain. His eyes were a good deal suffused before I commenced; his pulse not materially affected, but a little depressed. He continued sound asleep for fifteen minutes after the operation, and then awoke, complaining of nausea, which soon, however, wore off. Still being drowsy, we laid him upon the bed, where he continued to dose and wake for an hour. He complained two days after of the taste of the chloroform in his mouth and a slight pain in his breast; but these soon left him, and he is now doing well. He is the only one to whom I have given it who would prefer to undergo the same operation again without chloroform. In this case I made use of a small glass inhaler, invented by Rushton & Clarke of New York city. I think that *it* was the cause of the cough and other symptoms of pulmonary irritation of which he complained. I should certainly not use it again in any case. I can inhale the chloroform from a handkerchief without any discomfort, but with the inhaler my lungs were excessively irritated, and I could scarcely breathe.

Case VI.—Ann Contie, æt. 45, consulted me about a tumour which was developing itself rapidly in her left mamma, which presented all the characteristics of scirrhus; and as it pained her much and disabled her entirely, I advised its removal. The operation took place Tuesday, March 14th. She was a good deal agitated at the time, and much excited by the presence of the gentlemen who were there to assist me and see the effect of chloroform. Upon exhibiting this to the extent of between two and three drachms she became outrageous, and could with difficulty be held in her chair; and as her pulse was depressed, and she begged "no more of that," I thought it prudent to desist, and completed the operation without it. She screamed loudly at my incisions through the skin, but afterwards was perfectly quiet, so much so that some thought she was under the influence of chloroform; but this she denied, declaring she was suffering much. Nothing worthy of remark occurred subsequently. One reason why so much less chloroform is requisite in obstetrics than in surgery, and why that smaller quantity acts so much more rapidly and pleasantly, is the different state of the mind in the two cases; the instinctive horror of the knife, as well as the shrinking from the untried experiment of losing your senses, places the mind in a state of excitement difficult to be overcome. In labour, the sufferer feels the pain present and increasing, and generally begs you to give her ease. She inhales with gladness, and besides this no unknown and curious faces disturb her privacy. The chamber of her confinement is sacred, and every thing conspires to render it the very reverse of the operating room.

I have found the greatest possible benefit from chloroform in colic. The first time I used it in this distressing complaint was peculiarly gratifying. I found the patient almost exhausted with violent cramp colic, after having taken a good dose of castor oil with laudanum, and the usual domestic remedies having been applied without any effect. I then used the chloroform, and with such success that in two minutes he lay perfectly quiet in his bed, and though not asleep, yet entirely free from pain. I directed hot fomentations to be assiduously applied and a large warm injection, to be repeated, if necessary. He was so perfectly easy, however, that the family did not think it worth while to disturb him by any further remedies. I left him at 7 o'clock, P. M., and he continued thus till 10, when becoming a little uneasy, my remedies were tardily administered and the man soon became well. I have used it to procure sleep in a case of acute ulceration of the knee-joint, where the usual opiates had failed to procure a moment's ease for two days. In this case I administered it five different times, and each time with most happy results; refreshing sleep followed, and the case soon recovered. In painful diseases of all kinds, in anæmia with its neuralgias, in spasmodic asthma, &c., I have prescribed it for intelligent patients, directing ten or fifteen drops to be placed upon a handkerchief and gradually inhaled until a slight giddiness was the effect, and this repeated daily *pro re nata*, with the happiest results. I think, however, that in every case the lungs and heart should be carefully explored before its administration; any degree of congestion of the former or obstructive disease of the latter, especially dilatation, should forbid its use. In all cases, too, I consider it safest to have the fingers upon the pulse during its exhibition, and stimulants at hand, so that upon any faltering of the pulse, or any approach of danger, the use of it should be at once suspended and remedies used. Neither would an electro-magnetic machine in operation be an idle provision, as I have no doubt that had such a powerful stimulus to the heart been in readiness in the fatal cases reported, and at once resorted to, they would not have resulted so unfortunately. Pain itself is a stimulus, and its effect is directly as its severity up to a certain degree. This is one reason why I believe that chloroform is particularly safe in obstetrics. But a still greater reason for its general use in this department of the profession is, that a much less sound sleep is requisite, and a much smaller quantity of this powerful agent needed to effect the purpose. And as such cases are almost infinitely more frequent than cases of surgery, it is in midwifery that I believe chloroform will achieve its greatest triumphs and with the fewest accidents; with none, if proper precautions are used.

Besides these cases I have used it in several minor operations in surgery, and have witnessed its exhibition many times in the extraction of teeth, and hitherto have seen no more unpleasant results than a temporary nausea and emesis. In one case, when I did not wish or think proper to use it, but was urged, I consented to try it cautiously, but was soon compelled to stop, as it gave rise to unpleasant sensations. Upon the whole I should not countenance its exhibition in dentistry,—certainly never without the careful preparatory exploration of the chest by a competent physician. But even if it be used in the free and unguarded manner, as it has been hitherto, with so few fatal results distinctly traced to its influence, how small is that fatality compared with the loss of life from the use of steam in travelling? Those scores of accidents by which such a number of persons annually come to an awful death, do not prevent the public from using the steamboat and railroad car. Neither, *a fortiori*, as pain and agony are more important than mere time, should the two or three cases of death reported in the newspapers as occasioned by the use of chloroform, prevent the general careful use of this agent in midwifery and surgery.

### *Chloroform in Midwifery.*

Case I.—Feb. 20, 1848. Being called at five o'clock this morning to Mrs. C——, a healthy female, already in labour with her seventh child, after proper examination I concluded to try the effects of chloroform. I had already used this agent in a variety of surgical operations with success and without the least unpleasant consequence, and felt anxious to test it in this case. The os uteri was well dilated, the pains frequent and severe; and when I told her that I would put her to sleep so that she could have her baby without any pain, and without any danger either, she urged me to do so. Pouring thirty or forty drops upon my handkerchief, I

placed it about two inches from her face, telling her to open her mouth and breathe it in with long, deep inspirations, and after a breath or so I placed it nearer, till it finally rested loosely over the face. She soon showed by a few motions of the arm that she was under its influence; the pains seemed to be suspended, but using it less freely they came more and more frequently and forcibly, until in less than an hour after she commenced taking it the labor was over. When each pain seized her she caught hold of my hands, bearing down with all her force until it was over, when she lay perfectly still again. Once she partially awoke and begged me to put her to sleep again. When the head was passing the external organs, I had left her head, and she became partially sensible of the pain; but this was all she felt, as she told me, and that was not severe. She remained awake after this, but I was satisfied with my experiment. She had no headache or any unpleasant effect afterwards from the use of the chloroform, and mother and child have both been doing remarkably well to this time. Her pulse was perfectly natural and undisturbed throughout. I might mention that she vomited several times previous to taking the chloroform, but that stopped so soon as she fell under its influence.

Case II.—Mrs. S., of Washington, already the mother of eight children, in her confinement with each of which she had had exceedingly painful and protracted labours, was looking forward to her coming confinement with excessive anxiety and dread, when she heard of the use of chloroform in such cases and sent for me. After stating these facts to me, she asked if I thought she could take it with safety? I found her pulse full and strong, her health robust, with nothing in her past or present medical history to contra-indicate its use. Her labour had usually lasted from twelve to thirty-six hours, with irregular pains, which did not become effective till after venesection. She once had laboured under asthma, but now was free of it; and after an exploration of the heart and lungs, I advised her to make trial of the chloroform. To this she joyfully assented. I was called to her Wednesday night, March 1st, about midnight. She had been teazed with pretty sharp pains all day, but was sitting up; pulse 100, full, strong; pains irregular, but about every fifteen or twenty minutes. Upon examination I found the head high up and the os partially open, but rigid. She remained thus till 5 o'clock in the morning, when I found her pulse

110 and strong; the os a little more dilated, but still unyielding, and with an edge as if surrounded by a wire; pains irregular, but harder, and recurring every ten or fifteen minutes. She continued in this way till noon, when I bled her to the extent of a pint, and finding the neck of the womb more dilateable and the pains more frequent, I ruptured the membranes and gave her the chloroform. I used it in the same quantity and manner as in my first case, and after a few inspirations she said, "now I feel it, now I'm going, I'm gone." Soon after a pain seized her more severe than any hitherto, and she made great exertions to bear down. During the continuation of her labour she lay with composed features, as if in tranquil sleep at times, but generally talking to herself, and praying most devoutly, in beautiful language; looking about her calmly and speaking to some ladies, her friends, who were present; sometimes calling them by their right names and sometimes mistaking them for others; speaking to her husband, who held one of her hands, and frequently addressing me as the "good doctor," or as Dr. —— or Dr. ——. After each pain I removed the handkerchief from her mouth, but returned it freshly moistened just as each one returned again, and kept it so until it was over. They seemed to be encouraged by the chloroform—not at all delayed or enfeebled. The head continued to descend slowly, the anterior lip of the os still feeling thick and rigid, the pains becoming more and more violent, so much so that I feared some danger to the womb. After one very violent pain she exclaimed, "was not that splendid?" After another, "well, I've been in Heaven once, that's certain, but I don't know as I shall get there again." At length the head was born; I surrendered the handkerchief to one of the ladies, to attend to the child, and while the shoulders were passing she partially awoke, the chloroform having evaporated from the handkerchief; but upon moistening it freshly she fell asleep again. The child's head was a good deal elongated by the pressure, and it was some moments before it breathed, but it soon revived and cried well. In a few moments the placenta was expelled—the soiled clothes removed—a bandage placed around her, and she now awoke. This was at a quarter before 3, P. M. Her pulse had all the time been about 90, soft and good; her face natural; the bandage around her arm became loose in the violent efforts she made to bear down, and she lost nearly half a pint more of blood before it was secured.

She was left strictly to herself, without conversation, and quite comfortable. Visiting her in the evening I found the family much alarmed; Mrs. S. had lost nearly a quart of blood, as the nurse informed me, from the womb, and had nearly fainted, but had revived before I came. I found the pulse 110 and feeble; the bandage loosely applied over the abdomen, and the womb considerably enlarged. I at once reapplied the bandage as tightly as possible, and placed between it and the womb a small book to make additional pressure. At the time she was much better; said she knew nothing of her labour after she first took the chloroform, until the shoulders were passing, when she thought it was an after-pain. She described her sensations as delightful, and knew nothing of what she had said. I had forgotten to mention that she asked for water three or four times while asleep and drank heartily; of this, too, she had no recollection. She, and all those who watched her so anxiously, were delighted at the result, which they dreaded the more, as their fears as to the safety of using chloroform in any case had just been raised to the highest degree by the sad result of its use in the case of Mrs. Simmonds, of Cincinnati. At present they are all resolved to use it themselves when their time comes. Mrs. S. is urgent that another acquaintance should use it, who is daily expecting to be confined; and she says, also, that as soon as she gets up she will take it to have her teeth removed to make way for an artificial set. I mention these incidents to show how much confidence she has in its use. Since the first day she and her child have progressed remarkably well. She feels better, she says,—less sore than after any of her previous labours. Heretofore she has not slept for one or two nights after her accouchements, and has been bled about the third day after, in all; but she slept well the night after her labour this time, and I found her the next morning with a pulse at 90 and good; child nursing; no headache or sick stomach, or nervous excitement, and with a great deal of gratitude to me for converting a bed of bitter anguish into an Elysium. About an ounce of chloroform was used in each of the preceding cases.

Case III.—I was called at 5, P. M., March 9th, to Catherine Hawkins, mother of the little boy upon whom I operated (see Case 1st, Surgical Cases) for necrosis of the fibula. She was in labour with her seventh child. I found the head high up, the os well dilated; pains irregular and ineffective. A midwife had been with her since morning, and sent for me to see what was the matter. The pulse was natural; and, as all circumstances were favourable, I proceeded to rupture the membranes; and, pouring thirty drops of chloroform upon my handkerchief, I applied it gradually to her face. She fell under its influence in a few seconds, with no external symptom whatever. Soon a severe pain followed, and the head rapidly descended. Pain followed pain quickly, while she lay in a quiet sleep, the pulse perfectly unaffected; and, in fifteen minutes from its first exhibition, I was made aware of the birth of the child by its hearty cries. I afterwards reapplied the handkerchief once or twice, slightly moistened each time, to keep her asleep until after the placenta should pass. This followed in a few minutes. I then put the handkerchief in my pocket, and sat by her side with my fingers upon her pulse; a bandage was applied, the child washed and dressed, and still she slept on, as in the most pleasant sleep, and did not finally awake until near 6 o'clock. She then quietly remarked, "I've had a good nap, and feel better;" and dozed again. I did not disturb her, as the nurse told me she had not slept any the night before, and her pulse was, all the while, perfectly natural; in fact I could not perceive that the chloroform had the slightest effect upon the pulse in any way. She soon woke again with the same remark, when I directed the nurse to bring the child to her; she seemed much affected when I remarked, "It was one of the neighbour's, it could not be her's." "I'm sorry," was the reply; I thought it was mine; but I don't feel any pain; *it is mine*, but I didn't feel any pain: how strange!" I left her, with strict orders that she be not disturbed, but to let her sleep as long as she would. In this case not more than fifty drops of chloroform were used in all. Since this she has rapidly recovered; on the third day she sat up, and has been doing so since. In her previous confinements she has been kept in bed a week or more, and has always had more or less pain; this time she says she feels perfectly well, and not at all as if she had been confined.

In reviewing the preceding cases, I would remark, that the chloroform I first used gave rise to more unpleasant effects, and it required more to soporize the patients, than that which I used in my latter cases. I am confident that very many of the unpleasant sequelæ of chloroform depend upon its impurities. I was struck by the fact that so much more was requisite to tranquillize my first patients than Dr. Simpson found sufficient. That which I am using now seems genuine. Too much attention to this point cannot be paid. All inhalers are worse than useless; I believe them dangerous. The handkerchief, as used by Prof. Simpson, is the best inhaler. Cæteris paribus, chloroform is safest in obstetrics; and I would use it in *all* cases where there was no positive counter-indication.

